# Case report: A case of rare metastasis of gastric cancer to the axillary lymph node metastasis treated with combination immunotherapy

**DOI:** 10.3389/fimmu.2024.1331506

**Published:** 2024-02-09

**Authors:** Jin Wang, Yu Cheng, Yulin Wang, Hengxin Liu, Shuang Wu, Guangwei Tian, Jinglei Qu, Xiujuan Qu

**Affiliations:** ^1^Department of Medical Oncology, The First Hospital of China Medical University, Shenyang, China; ^2^Provincial Key Laboratory of Anticancer Drugs and Biotherapy of Liaoning Province, The First Hospital of China Medical University, Shenyang, China; ^3^Clinical Cancer Research Center of Shenyang, The First Hospital of China Medical University, Shenyang, China; ^4^Department of Radiation Oncology, The First Hospital of China Medical University, Shenyang, China

**Keywords:** case report, gastric cancer, axillary lymph, immunotherapy, Epstein-Barr virus

## Abstract

Lymph node (LN) metastasis is a common mode of metastasis in advanced gastric cancer (GC), while axillary LN metastasis infrequently occurs in GC. There are few reports on this rare type of metastasis – especially its clinicopathological features – and systemic treatment are unclear. We describe a case of GC with extensive metastasis, including the rare axillary LN metastasis. The patient achieved partial response of optimal efficacy, who was treated with combination immunotherapy as second-line treatment for nearly two years. The potential mechanisms were revealed by clinical and immune characteristics, such as high expression of PD-L1, high tumor mutational burden (TMB-H), Epstein-Barr virus (EBV) positive and CD8^+^ tumor-infiltrating lymphocyte positive.

## Introduction

Based on current global cancer statistics, gastric cancer (GC) continues to have a significant impact on a global scale, with China being identified as one of the countries with high rate of incidence thereof. In fact, both the incidence and mortality rates of GC in China rank third among all malignant tumors (1). Recent advances have greatly enhanced the level of prevention, screening, diagnosis, and treatment for GC. Nevertheless, the insidious nature of GC often leads to late-stage diagnoses, with the majority of patients who have already progressed to the advanced or locally advanced stages. The presence of an extensive network of lymphatic vessels in the stomach contributes to the prevalence of lymph node (LN) metastasis in GC. The incidence of LN metastasis of advanced GC was 52.2%-82.6%, being mainly regional LN metastasis (2, 3). Distant LN metastasis of GC frequently occurred in the left supraclavicular LN, mainly through the thoracic duct. However, the occurrence of axillary LN metastasis in GC is infrequent. Treatment options for patients with advanced stage GC are limited, with chemotherapy combined with targeted therapy being the conventional approach. Recent studies have found significant advancements in the use of immunotherapy for treating advanced GC, leading to long-term survival benefits for select patients. The immune checkpoint inhibitor PD-1 monoclonal antibody has been approved as a third-line treatment for advanced GC, while its combination with chemotherapy has emerged as a new standard for the first-line treatment of advanced metastatic GC (4, 5).

We report a case of GC with extensive metastasis, including the rare axillary LNs metastasis, in which combination immunotherapy was prescribed as second-line treatment to good effect.

## Case description

**Figure 1:**
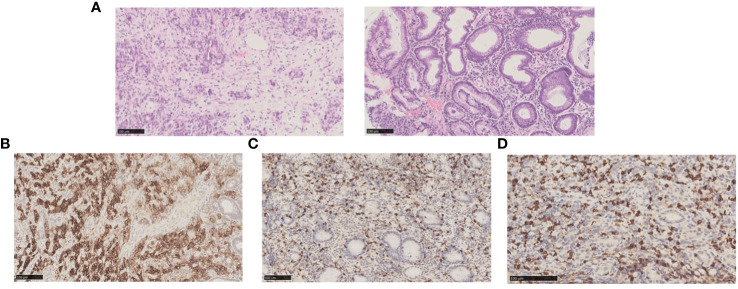
Biopsy and pathology examination of gastric and axillary LNs before chemotherapy. **(A)** Hematoxylin-eosin staining pictures of the gastric (left) and axillary LNs (right) demonstrated adenocarcinoma, indicating that LN was a metastasis of GC. **(B)** Immunohistochemical staining indicated gastric tumor tissue expressed PD-L1. **(C)** Immunohistochemical staining showed gastric tumor tissue expressed CD8. **(D)** Immunohistochemical staining showed gastric tumor tissue expressed EBV.

A 41-year-old male patient with abdominal distension accompanied by eating obstruction, was admitted to our hospital in July, 2020. The patient was diagnosed as gastric poorly differentiated adenocarcinoma by gastroscopy and pathological examinations (**Figure 1A**). Enhanced computed tomography (CT) demonstrated that thickening of the stomach wall on the lesser curvature of the stomach body, multiple enlarged LNs in the abdominal cavity, GC penetrating the serous membrane with extensive LN metastasis, and multiple enlarged LNs in the left neck and armpit. Biopsy of left axillary LN showed metastatic adenocarcinoma of gastric origin (**Figure 1A**). A bone scan illustrated multiple bone metastases throughout the body. The clinical stage was cT4N_+_M1 (axillary LN, bone, and abdominal metastasis) according to AJCC Version 8.0.

**Figure 2:**
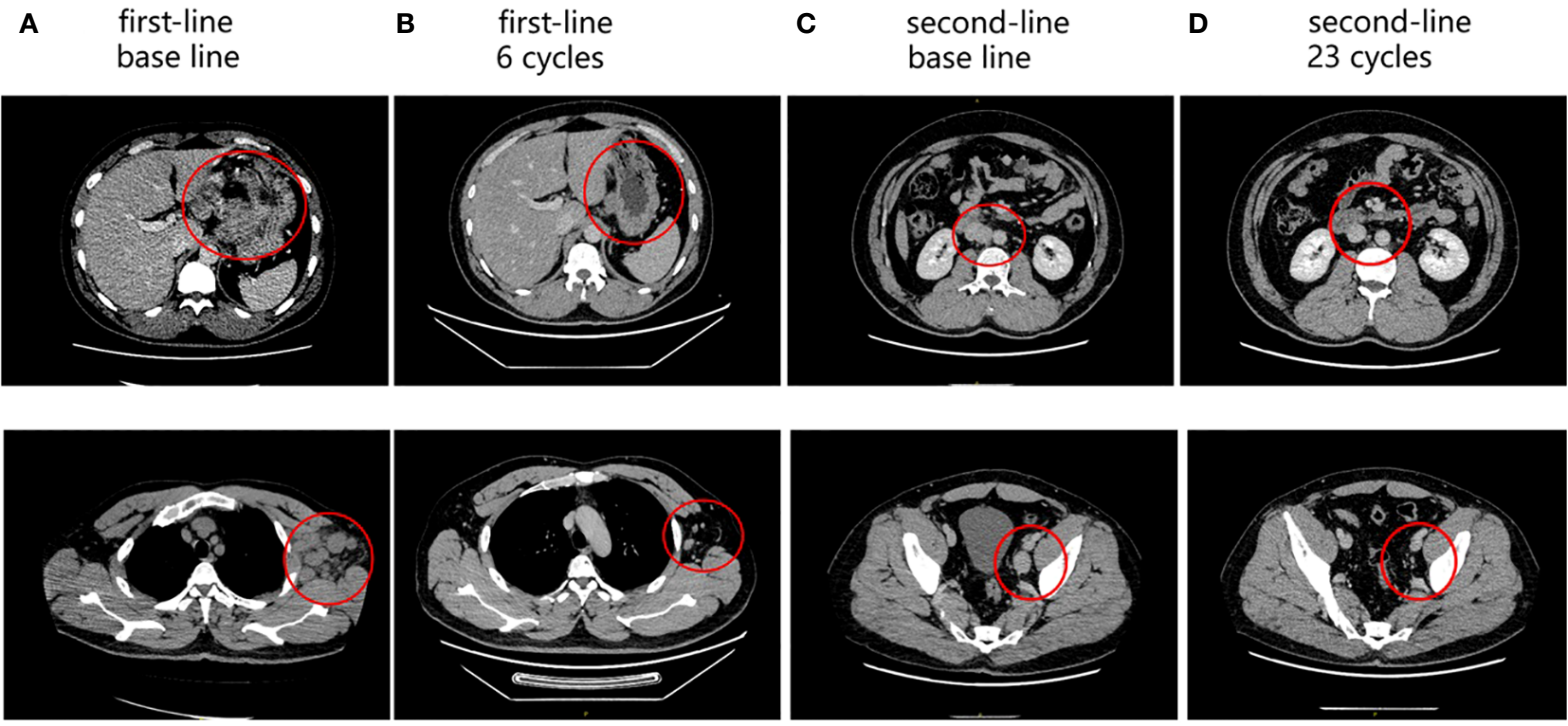
Enhanced abdominal CT during the follow-up period. **(A)** Baseline of first-line treatment: perigastric LNs (up); axillary LNs (down). **(B)** After six cycles of first-line treatment: perigastric LNs (up); axillary LNs (down). **(C)** Baseline of second-line treatment: retroperitoneal LNs (up); pelvic LNs (down). **(D)** After 23 cycles of second-line treatment: retroperitoneal LNs (up); pelvic LNs (down). The red circles represent the target lesion.

As first-line chemotherapy, the patient was treated with a CAPOEX regimen (oxaliplatin 130 mg/m^2^ once on day 1 plus capecitabine, 1000 mg/m^2^ twice a day on days 1–14) for eight cycles (3 weeks per cycle) and single capecitabine maintenance therapy as follows. The best effect was partial response (PR) which is reflected in the reduction of hepatic hilar and parietal LN metastases (**Figure 2**). Additionally, the patient tested positive for PD-L1 (combined Positive Score [CPS] > 70%), CD8 T-cell lymphoma and Epstein-Barr virus (EBV) positive in gastric tissue (**Figures 1B–D**). The immunostaining for human epidermal growth factor Type 2 (HER2) was negative and microsatellite instability (MSI) tests indicated microsatellite stability (MSS). A tumor mutational burden (TMB) of 15.04, 11.81, and 21.48 mutations/Mb present in gastric tissue, axillary LN, and blood, separately, was categorized as high TMB (TMB-H) by next-generation sequencing results.

Disease progression is manifest as retroperitoneal and parietal pelvic LN enlargement. The patient was enrolled in a clinical study of albumin-bound paclitaxel (125 mg/m^2^ on day 1 and day 8) combined with apatinib (250 mg once a day) and camrelizumab (200 mg on day 1) for seven cycles (3 weeks per cycle) as second-line treatment. The best curative effect was PR (**Figure 2**). However, the patient suffered from hypertension, increase of G2 bilirubin, increase of G3 aminotransferase, and G3 myelopathic depression. After the 7^th^ cycle, the patient withdrew from the clinical trial due to the recurrence of G3 aminotransferase elevation. The patient was then treated with apatinib (250 mg daily for 2 days discontinued for 1 day) combined with camrelizumab (200 mg on day 1) for seven cycles (3 weeks per cycle). Apatinib was adjusted to fruquintinib (5 mg daily on days 1-21) due to the recurrence of G3 liver dysfunction. The trends of liver function indexes were shown in **Supplementary Table 1**. The patient received camrelizumab and fruquintinib for one year without abnormal liver function indicators above G2.

**Figure 3:**
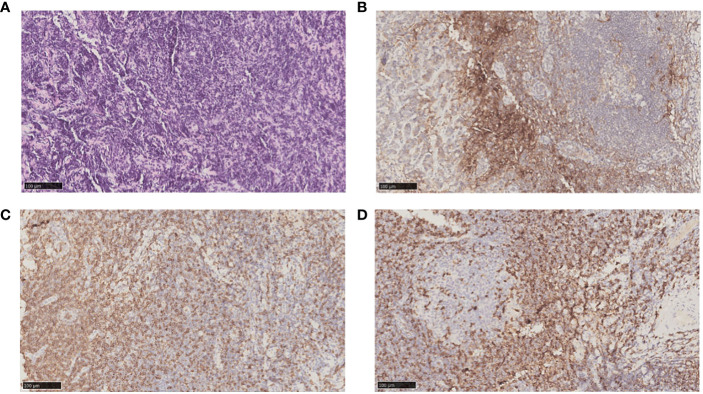
Biopsy and pathology examination of left supraclavicular LNs before chemotherapy after second-line treatment progresses. **(A)** Hematoxylin-eosin staining pictures of left supraclavicular LNs. **(B)** Immunohistochemical staining showed left supraclavicular LNs expressed PD-L1. **(C)** Immunohistochemical staining showed left supraclavicular LNs expressed CD8. **(D)** Immunohistochemical staining showed left supraclavicular LNs expressed CD3.

**Figure 4:**
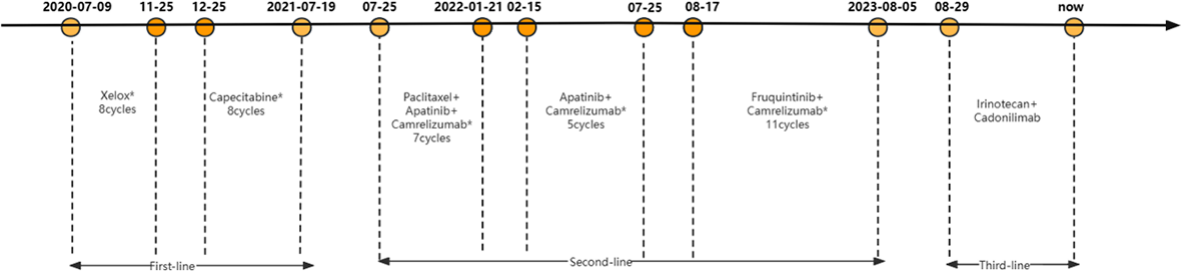
Timeline of the treatment of this patient.

After nearly two years of combination immunotherapy, the left supraclavicular and axillary LNs were enlarged. The biopsy of left supraclavicular LN showed metastatic gastric adenocarcinoma (**Figure 3A**). The result of PD-L1 in tumor proportion scoring (TPS) was 30% and CPS was 65 (**Figure 3B**). CD3 and CD8 were positive sporadically. CD8/CD3 T-cell lymphoma was 80% (**Figures 3C, D**). Due to the efficacy of previous anti-PD-1 therapy, the third-line treatment is to use cardonilizumab (PD-1 and CTLA-4 inhibitors) and irinotecan chemotherapy. The patient’s overall survival has now reached 37 months (**Figure 4**).

## Discussion

This case revealed three important clinical issues. First, this was a rare case of metastasis to axillary LNs for GC. The primary metastatic sites of this patient were LNs, including rare axillary LNs and common supraclavicular and abdominal LNs, without liver or lung metastasis. Second, based on the aforementioned characteristics of the disease, the effective time of the patient who received the combination immunotherapy was nearly two years. Third, the patient had recurrent liver dysfunction during the course of combination therapy. However, no serious liver dysfunction occurred after apatinib was replaced by fuquitinib.

In clinical practice, metastatic lesions in the lower region of the stomach commonly spread to the subpyloric, subgastric, and paraortic LNs, whereas those in the upper region tend to metastasize to the para-pancreatic, para-cardiac, and superior gastric LNs. In advanced stages of the disease, metastasis may also occur in the superior mediastinal LN. Additionally, the abdominal lymphatic circulation directly connects to the thoracic duct, enabling cancer cells to potentially reach the left supraclavicular LN (6). However, axillary LN metastasis for GC was rare. The diagnostic challenge in this case was to identify axillary lymph nodes as GC metastases. The immunohistochemical (IHC) staining of axillary lymph node for CK7, SATB2 and CDX-2 was negative, which was the same as that of gastric tissue and left supraclavicular lymph node. The IHC staining of CDX-2 was negative in axillary lymph node and left supraclavicular lymph node, and minority positive in gastric tissue (**Supplementary Figure 1**). Combined with the medical history, both the left axillary lymph nodes and the left supraclavicular lymph nodes were considered as GC metastases. To our knowledge, only two documented cases have been reported in detail. Zhu reported a case of left axillary LN mass that grew faster after one month of radical total gastrectomy and then was considered as GC metastasis by axillary LN biopsy. This case refused further treatment and died 11 months after axillary LN metastasis (7). Kobayashi reported a case of isolated left axillary metastasis 21 months after radical distal gastrectomy and underwent radical axillary LN dissection without tumor recurrence in 1-year follow-up (8).

The common routes of metastasis for GC encompass direct invasion, LN metastasis, hematogenous metastasis, and transcoelomic spread. The LN metastasis of GC mainly occurs gradually along the LN drainage pathway, but some patients show cross-regional distant LN jump metastasis. Previous case report has revealed the possible mechanism of axillary LN metastasis in GC (7). The possible mechanism of the axillary LN metastasis in our case might be the combination of LN metastasis and hematogenous metastasis. Firstly, the lymphatic drainage from axillary LNs comes from subcutaneous or intercostal lymphatic vessels in the chest wall (8). The celiac LNs can be drained directly to the LNs of chest wall and then to the thoracic LNs in some cases (9). Secondly, the tumor cells invade the thoracic duct, enter the blood circulation and then enter the left subclavian lymphatic vessel via the left subclavian vein. And with the direction of axillary LN drainage counter-current, axillary LN metastasis occurred. Thus, this case presented with left supraclavicular and left axillary LN metastasis.

Relevant studies have found that immunotherapy has made a breakthrough in the treatment of metastatic GC (4, 10). However, in the treatment of GC, the effective rate of immune checkpoint inhibitors (ICIs) is only about 10%, and the research hotpot is to detect biomarkers to explore the efficacy prediction indicators of ICIs (11–13). Several studies have reported the signature of tumor immune microenvironment, such as the PD-L1 expression and tumor infiltrating lymphocytes (TILs), could be related with a better response to immunotherapy (14). KEYNOTE-158 showed that solid tumor patients with tissue TMB-high (tTMB-H,TMB ≥ 10 mutations/Mb) benefited significantly from immunotherapy (15). Previous studies have shown that the objective response rat to PD-1 inhibitors in EBV positive GC was about 25%, much greater than that in EBV negative GC (11, 16–18). Qiu reported that EBV positive GC exhibited an inflamed-immune phenotype with increased T-cell and B-cell infiltration by dynamic single-cell mapping (19). EBV positive GC showed inflammatory response from neo-antigens, and immunotherapy triggered clonal reactivation and reactivation of effector T cells, which improved the therapeutic response (19, 20). In this case, the patient exhibited multiple factors for which immunotherapy may be effective, including high expression of PD-L1, TMB-H (including tTMB-H and blood TMB-H), EBV positive and CD8+ TILs positive. These are probably the main reasons why this patient has benefitted from the efficacy of immunotherapy for two years, but it is difficult to determine the specific factor responsible for this. Moreover, liver metastasis is often considered as a poor response-predictor of anti-PD-1 therapy (21). This patient did not develop liver metastasis during the whole treatment, which may also be one of the reasons why the patient received better results from immunotherapy. The patient was only 41 years old when he was first diagnosed, was the main source of income for his family, and the good treatment results allowed him to work and live normally. The patient now has an overall survival time of 37 months, mainly due to the benefit of nearly two years of combination immunotherapy.

Drug-induced liver injury (DILI) is a major concern in tumor treatment, especially in the combination of chemotherapy, immunotherapy and targeted therapy. In the initial detection stage of liver injury, it is difficult to determine which specific drug is responsible for the abnormal liver function. In this case, the liver function of this patient can be restored after the treatment of liver protection alone, so the possibility of immune liver injury is considered to be less. In addition, the patient repeatedly experienced abnormal liver function when only combined chemotherapy and targeted drugs were adopted, so it was considered that DILI may be more correlated with chemotherapy and vascular endothelial growth factor receptor-tyrosine kinase inhibitors (VEGFR-TKI). Interestingly, after switching the VEGFR-TKI from apatinib to fuquitinib, the patient did not develop liver injury above G2. Both apatinib and fuquitinib were metabolized by cytochrome P450 isoenzyme 3A4 (CYP3A4), and inhibited VEGFR. The specific receptors of above two drugs are different. For example, apatinib mainly acts on VEGFR-2, while fuquitinib acts on VEGFR 1-3. The pathogenesis of DILI is complex and often the result of multiple mechanisms of action successively or jointly. The exact mechanism acting in this case may be an issue for future consideration.

## Conclusion

The present study reports a case of GC with multiple metastases including rare axillary LN metastasis and showed partial response to nearly two years of combination immunotherapy as second-line therapy. Pathological examination of abnormally enlarged LNs is still the best approach to identify the primary tumor. More research is needed to determine which treatment is more conducive to improving the prognosis of GC patients with axillary LN metastasis.

## Data availability statement

The original contributions presented in the study are included in the article/**Supplementary Material**. Further inquiries can be directed to the corresponding authors.

## Ethics statement

The studies involving humans were approved by the Ethics Committee of the First Hospital of China Medical University. The studies were conducted in accordance with the local legislation and institutional requirements. The participants provided their written informed consent to participate in this study. Written informed consent was obtained from the individual(s) for the publication of any potentially identifiable images or data included in this article.

## Author contributions

JW: Writing – review & editing, Writing – original draft. YC: Writing – review & editing, Visualization. YW: Visualization, Writing – review & editing. HL: Visualization, Writing – review & editing. SW: Visualization, Writing – review & editing. GT: Writing – review & editing, Conceptualization. JQ: Writing – review & editing, Supervision, Visualization. XQ: Supervision, Visualization, Writing – review & editing.

## Supplementary material

Supplementary Figure 1 Hematoxylin-eosin and immunohistochemical staining of gastric and axillary lymph node. **(A)** Hematoxylin-eosin and immunohistochemical staining pictures of the gastric. Immunohistochemical staining showed that gastric was negative for CK7 and SATB2, minority positive for CDX-2. **(B)** Hematoxylin-eosin and immunohistochemical staining pictures of the axillary lymph node. Immunohistochemical staining showed that axillary lymph node was negative for CK7, SATB2 and CDX-2.
