# Supplementary figures and images for: Case report: A case of rare metastasis of gastric cancer to the axillary lymph node metastasis treated with combination immunotherapy
Source: Front Immunol. 2024 Feb 9;15:1331506. doi: 10.3389/fimmu.2024.1331506 (PMC10884146; doi:10.3389/fimmu.2024.1331506)

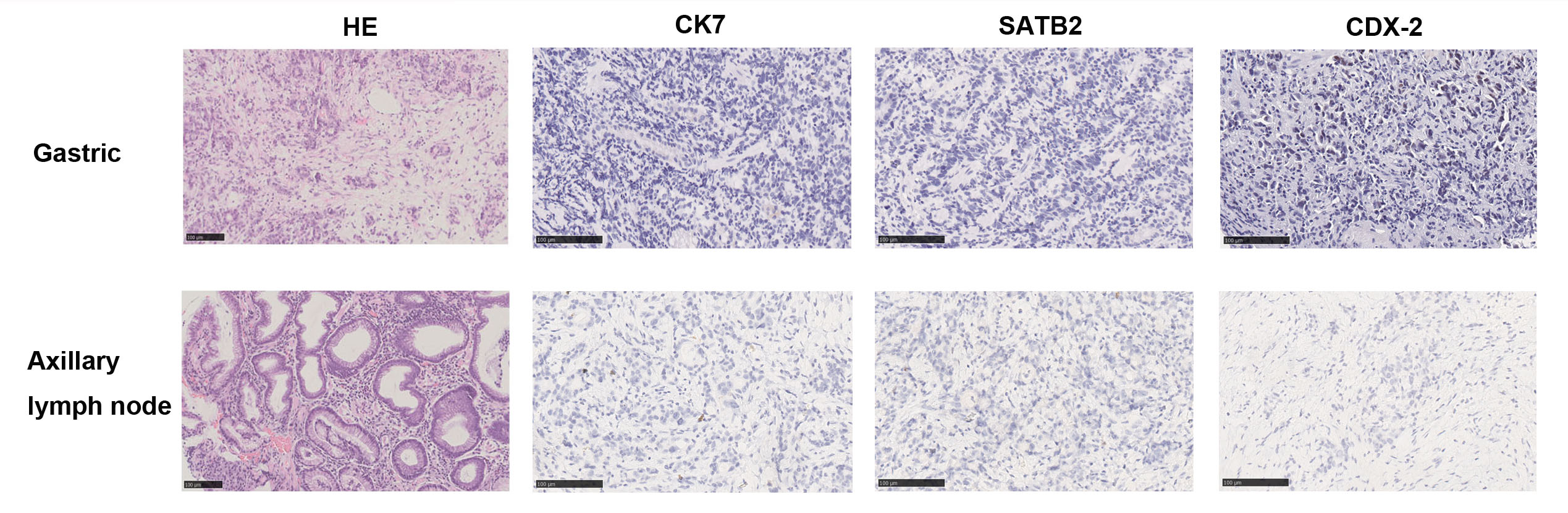

Supplement: Supplementary Figure 1 — Hematoxylin-eosin and immunohistochemical staining of gastric and axillary lymph node. (A) Hematoxylin-eosin and immunohistochemical staining pictures of the gastric. Immunohistochemical staining showed that gastric was negative for CK7 and SATB2, minority positive for CDX-2. (B) Hematoxylin-eosin and immunohistochemical staining pictures of the axillary lymph node. Immunohistochemical staining showed that axillary lymph node was negative for CK7, SATB2 and CDX-2. [file Image_1.jpg]
