# Supplementary material for: Case report: A case of rare metastasis of gastric cancer to the axillary lymph node metastasis treated with combination immunotherapy
Source: Front Immunol. 2024 Feb 9;15:1331506. doi: 10.3389/fimmu.2024.1331506 (PMC10884146; doi:10.3389/fimmu.2024.1331506)
Supplement: Supplementary file 2 [file Table_1.docx]

Supplementary Table 1. The trends of liver function indexes during the second line combination immunotherapy.

|  | 2021.8.19  (C1D1) | 10.8  (C3D1) | 10.28  (C4D1) | 11.19  (C5D1) | 12.9  (C5D21) | 12.13 | 12.23  (C6D1) | 12.31  (C6D8) | 2022.  1.3 | 1.14  (C7D1) | 1.21  (C7D8) | 2.14^a^ | 3.8 | 5.24 | 7.25 | 8.16^b^ | 2023.1.4 | 3.17 | 8.14 |
| --- | --- | --- | --- | --- | --- | --- | --- | --- | --- | --- | --- | --- | --- | --- | --- | --- | --- | --- | --- |
| ALT | **66↑**  **(G1)** | **55↑**  **(G1)** | 39 | **91↑**  **(G1)** | **74↑**  **(G1)** | 44 | 43 | **124↑**  **(G1)** | **68↑**  **(G1)** | 41 | **114↑**  **(G1)** | **51↑**  **(G1)** | **77↑**  **(G1)** | **73↑**  **(G1)** | **105↑**  **(G1)** | 38 | 47 | 34 | 36 |
| AST | **44↑**  **(G1)** | 32 | 33 | **60↑**  **(G1)** | **134↑**  **(G2)** | **91↑**  **(G1)** | **77↑**  **(G1)** | **275↑**  **(G3)** | **89↑**  **(G1)** | **69↑**  **(G1)** | **202↑**  **(G3)** | **50↑**  **(G1)** | **128↑**  **(G2)** | **77↑**  **(G1)** | **212↑**  **(G3)** | 38 | 37 | 30 | 32 |
| TBIL | 19 | 11 | 10 | 11 | 24 | 24 | 10 | 24 | 16 | 10 | 24 | 10 | **27↑**  **(G1)** | 17 | **31↑**  **(G1)** | 14 | 14 | 12 | 13 |
| DBIL | 4 | 3 | 3 | 3 | **13↑**  **(G2)** | **14↑**  **(G2)** | 5 | **14↑**  **(G2)** | **11↑**  **(G1)** | 5 | **13↑**  **(G2)** | 4 | **15↑**  **(G2)** | 4.4 | 6.5 | 4 | 4 | 3 | 3 |

ALT: alanine aminotransferase, U/L; AST: glutamic oxaloacetic transferase, U/L; DBIL: direct bilirubin, umol/L; TBIL: total bilirubin, umol/L.

The grading criteria for ALT or AST levels (CTCAE 5.0): G1(1to 3 times the upper limit of normal), G2(3 to 5 times the upper limit of normal), G3(5 to 20 times the upper limit of normal). The grading criteria for TBIL or DBIL levels (CTCAE 5.0): G1(1 to 1.5 times the upper limit of normal), G2(1.5 to 3 times the upper limit of normal), G3(3 to 10 times the upper limit of normal).

^a^ Apatinib+PD-1 inhibitor.

^b^ Fruquintinib+PD-1 inhibitor.
